# Which Anthropometric Index Best Signals 10‑Year Cardiovascular Risk? A Sex‑Stratified Analysis From the Shiraz Heart Study

**DOI:** 10.1002/clc.70524

**Published:** 2026-11-09

**Authors:** Mohammad Javad Zibaeenezhad, Alireza Arzhangzadeh, Mehrab Sayadi, Alireza Khodadadiyan, Melika Golmohammadi, Zahra Hooshanginezhad, Ebrahim Rahimi, Maryam Jafari, Mehdi Bazrafshan, Nader Parsa, Iman Razeghian‐Jahromi, Roozbeh Narimani‐Javid, Seyyed Saeed Mohammadi, Fatemeh Zibaeenejad, Davar Aldavood, Hamed Bazrafshan drissi, Masih Jamshidi

**Affiliations:** ^1^ Cardiovascular Research Center Shiraz University of Medical Sciences Shiraz Iran; ^2^ School of Medicine Shahid Beheshti University of Medical Sciences Tehran Iran; ^3^ Mamasani Higher Education Complex for Health Shiraz University of Medical Sciences Shiraz Iran; ^4^ Student Research Committee, School of Medicine Anzali International Campus, Guilan University of Medical Sciences Rasht Iran; ^5^ Research Center for Advanced Technologies in Cardiovascular Medicine, Cardiovascular Diseases Research Institute Tehran University of Medical Sciences Tehran Iran

**Keywords:** adiposity tissues, anthropometric indices, atherosclerotic cardiovascular disease, cardiovascular, obesity

## Abstract

**Background:**

Atherosclerotic cardiovascular disease (ASCVD) remains a major global cause of morbidity and mortality, with obesity as a critical contributor. Traditional measures like body mass index (BMI) do not fully capture fat distribution or composition. Anthropometric and fat indices such as body fat (BF), may offer greater precision in ASCVD risk assessment.

**Methods:**

We analyzed data from 1106 university individuals (26−72 years). The primary analysis included adults 40−70 years (*n* = 761), for whom 10‐year ASCVD risk was estimated using the 2019 ACC/AHA Pooled Cohort Equations (PCE). We modeled continuous risk using sex‐adjusted linear regression with each index entered separately and We tested Index × Sex interactions. Discrimination for elevated risk (≥7.5%) was assessed with ROC/AUC. A secondary analysis in participants <40 years assessed discrimination for a composite high risk‐factor burden endpoint (≥2 abnormalities among BP, lipids, smoking, diabetes, BMI).

**Results:**

The median (IQR) of ASCVD risk score was 1.33 (0.55−3.77), significantly higher in men than women (2.67 vs. 0.65; *p* < 0.001). Most participants (73.1%) were classified as low‐risk, though men had greater representation in higher‐risk categories. Furthermore, BF, Waist‐to‐height ratio (WHtR), and Waist‐to‐hip ratio (WHR) showed the strongest positive associations with continuous PCE risk, whereas Fat‐free mass (FFM) was inversely associated (*p* < 0.001). For discriminating ≥7.5% risk, WHR performed best, BF and WC were modest and WHtR and BMI provided little to no discriminative ability.

**Conclusion:**

Anthropometric indices such as WC, WHR, and BF are valuable discriminators of ASCVD risk, surpassing BMI in risk assessment and stratification.

## Graphical abstract

In adults aged 40−70 years, body fat percentage, waist‐to‐height ratio, and waist‐to‐hip ratio were the strongest correlates of elevated 10‐year ASCVD risk, while fat‐free mass showed an inverse association. In younger adults, central adiposity and body fat measures were most strongly associated with higher ASCVD risk.

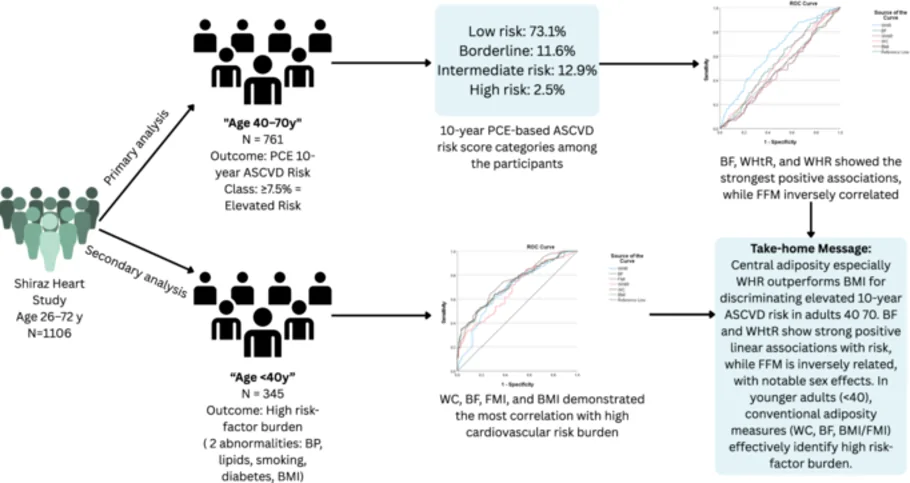

**Abbreviations**
ABSI: a body shape index
ASCVD: atherosclerotic cardiovascular disease
BAI: body adiposity index
BF: body fat
BMI: body mass index
BP: blood pressure
CAD: coronary artery disease
FFM: fat‐free mass
FFMI: fat‐free mass index
FM: fat mass
FMI: fat mass index
HC: hip circumference
HDL‐C: high‐density lipoprotein Cholesterol
LAP: lipid accumulation product
LDL‐C: low‐density lipoprotein Cholesterol
ROC: receiver operating characteristic
SHS: Shiraz Heart Study
TG: triglyceride
VAI: visceral adiposity index
WC: waist circumference
WHR: waist‐to‐hip ratio
WHtR: waist‐to‐height ratio

## 1 Introduction

Atherosclerotic cardiovascular disease (ASCVD) remains a leading cause of morbidity and mortality worldwide, driven by the progressive accumulation of lipid‐rich plaques in arterial walls, which can lead to myocardial infarction, stroke, and peripheral artery disease [1]. ASCVD affects over 120 million adults in the United States alone and is a significant health burden globally [2]. Furthermore, recent investigations into global trends in ASCVD have revealed an increasing number of patients suffering from cardiovascular disease (CVD) worldwide. More than half of these cases are attributed to atherosclerotic conditions, particularly ischemic heart disease, highlighting a concerning upward trend in CVD prevalence over recent decades [3]. While age‐adjusted mortality rates for ASCVD have decreased globally, the absolute number of deaths is increasing due to population growth and ageing [4].

The risk of developing ASCVD is influenced by a complex interplay of genetic, environmental, and lifestyle factors, among which obesity plays a pivotal role. Body mass index (BMI) has long been utilized as a simple anthropometric measure to categorize individuals based on weight relative to height and is widely accepted as a proxy indicator of overall body fatness [5]. However, the relationship between BMI and ASCVD risk is nuanced and may not fully capture the heterogeneity of adiposity distribution and its impact on cardiovascular health.

While BMI provides a general estimate of obesity, it does not distinguish between fat and lean body mass or account for the distribution of adipose tissue, which is increasingly recognized as a critical determinant of cardiovascular risk [6, 7, 8, 9]. In contrast to BMI, anthropometric indices such as waist‐to‐height ratio (WHtR) and waist‐to‐hip ratio (WHR) have been shown to better capture the complexities of visceral adiposity and abdominal obesity while offering simpler and more practical clinical assessment methods [10, 11, 12]. From a preventive cardiology perspective, waist‐based measures are particularly attractive because they are inexpensive, readily obtainable in routine clinical practice, and do not require laboratory testing or advanced imaging. Their simplicity may facilitate early risk identification and repeated assessment over time, potentially allowing clinicians to recognize adverse adiposity patterns before the development of overt cardiovascular disease [13]. Emerging evidence suggests that the location and type of adipose tissue—whether subcutaneous, visceral, epicardial, or perivascular, are more directly correlated with metabolic dysfunction and ASCVD risk than BMI alone [14]. While BMI is a useful measure of adiposity for populations, studies indicate it should be accompanied by body shape indices like waist circumference (WC) or WHR to identify high‐risk patterns of body fat (BF) distribution in overweight/obese patients [11, 15]. Similarly, assessing mortality risk with fat mass (FM) is more clinically significant than using BMI, which includes muscle mass that actually helps reduce cardiovascular risk [16].

Visceral adiposity, in particular, is associated with a higher prevalence of metabolic syndrome, insulin resistance, and inflammation, all of which contribute to atherosclerotic progression and subsequent cardiovascular events [15]. Adipose tissue acts as an active endocrine organ that secretes various bioactive substances, such as adipokines, cytokines, and hormones, which influence systemic inflammation, endothelial function, and lipid metabolism, thereby modulating ASCVD risk [17]. For example, excess visceral fat is linked to higher levels of pro‐inflammatory cytokines like interleukin‐6 (IL‐6) and tumor necrosis factor‐alpha (TNF‐α), which have been implicated in endothelial dysfunction and plaque instability [18].

Given the complexity of adiposity's role in cardiovascular health, there is growing interest in refining ASCVD risk assessment tools to incorporate measures of fat distribution and adipose tissue function. Current risk models primarily focus on traditional risk factors such as age, sex, smoking status, blood pressure, cholesterol levels, and diabetes mellitus but may benefit from the inclusion of more nuanced indicators of obesity‐related risk, such as WC, WHR, or direct measures of visceral fat through imaging modalities [19].

This study aims to explore the roles of anthropometric indices and various fat adiposity tissues in the context of ASCVD risk. Although similar studies discussed the efficacy of anthropometric indices on ASCVD risk, but none of them investigated the prognostic value of fat distribution indices [11, 20]. We examined the current understanding of how different types of adipose tissue and anthropometric indices contribute to atherosclerosis, discuss the limitations of using BMI as a classic anthropometric index for cardiovascular risk, and highlight potential strategies for incorporating adiposity measures into ASCVD risk assessment models. Additionally, we assessed sex‐specific differences in these associations to provide insights for tailored prevention strategies.

## 2 Methods and Materials

### 2.1 Study Design and Sample

This cross‐sectional study was designed based on Shiraz Heart Study (SHS) data from employees at Shiraz University and their spouses, which has recruited 1106 university employees between 26 and 72 years old as participants since 2017. This study was a complementary part of a larger study called the SHS, and the details of which are given in the SHS protocol study [21, 22]. Census sampling methods were used to recruit, and participants' information was collected through interviews and health system records. The data include socio‐demographic and anthropometric data, as well as data on diet and lifestyle habits and biochemistry (glucose and lipid profile). Participants with incomplete data were excluded from the study. This study was approved by the Shiraz University of Medical Sciences ethics committee (No: IR.SUMS.MED.REC.1401.568).

### 2.2 Clinical, Anthropometric, and Biochemical Parameters Assessment

All subjects were examined for hematochemical and clinical parameters. The lipid profile (Triglyceride [TG], Low‐Density Lipoprotein cholesterol [LDL‐C], Total Cholesterol, and High‐Density Lipoprotein cholesterol [HDL‐C]) were measured in fasting blood samples. Serum HDL‐C and LDL‐C levels were measured using direct enzymatic assay methods, while total cholesterol and TG concentrations were assessed using enzymatic colorimetric methods based on the cholesterol oxidase‐phenol aminophenazone (CHOD‐PAP) technique. Commercial assay kits manufactured by Biorexfars and Delta. DP were utilized according to the manufacturers' instructions. Biochemical analyses were performed using an automated clinical chemistry analyzer under standardized laboratory conditions, with routine internal quality control procedures applied throughout the study period [23, 24]. Additionally, the systolic and diastolic blood pressures were examined after 10 min of rest and in a sitting position with an OMRON sphygmomanometer [25, 26]. Three readings were obtained 1−2 min apart using an appropriately sized cuff, and the mean of the three measurements was used for analysis. The current smoking and diabetes status were reported based on medical history with “yes” or “no.”

Furthermore, we primarily estimated 10‐year ASCVD risk using the 2019 American College of Cardiology and the American Heart Association (ACC/AHA) Pooled Cohort Equations (PCE) for participants aged 40−70 years, with inputs such as age, sex, race, total cholesterol, HDL‐C, systolic blood pressure, antihypertensive treatment status, diabetes, and smoking [27]. The risk categories were established according to the clinical significance and the levels at which therapeutic measures needed to be taken to lower the risk of cardiovascular disease [28]. Participants < 40 years were considered for secondary analysis with a risk‐factor burden endpoint. Participants with more than two cardiovascular risk factors were considered as participants with high burden and abnormal risk factors.

The anthropometric indices, including weight, height, BAI (Body Adiposity Index), BMI, demi span, HC (Hip Circumference), WC, WHR, and WHtR were measured by an experienced clinician. It should be noted that weight and height were measured with light cloth and without shoes. Height was measured using a stadiometer that was fixed and calibrated in centimeters, accurate to the nearest 0.1 cm. Weight was measured using electronic scales that were accurate to the nearest 0.1 kg.

The functional definition of the mentioned variables is as follows:

BMI: the weight (kg) divided by the square of height (m) based on World Health Organization (WHO) cutoffs; overweight is defined as 25 < BMI ≤ 29.9 kg/m^2^, and obese is defined as BMI ≥ 30 kg/m^2^ [29].

WC: measuring the narrowest part of the abdomen or in the area between the lower margin of the last palpable rib and the iliac crest (minimum circumference). Elevated WC is considered more than 101 cm for men and more than 88 for women [30].

WHR: dividing WC (cm) by HC (cm).

WHtR: dividing WC (cm) by height (cm). Elevated WHtR is defined as > 0.5.

BAI: HC (cm) divided by (height [m])1.5 minus 18 [31].

BF (Body fat %): (1.20 × BMI) + (0.23 × Age)−(10.8 × Sex) − 5.4 [32]

(Sex: Male = 1, Female = 0)

FFM (Men): (0.407 × Weight [kg]) + (0.267 × Height [cm])−19.2 [33, 34]

FFM (Women): (0.252 × Weight [kg]) + (0.473 × Height [cm])−48.3 [33, 34]

FFM (If BF already known): Body Weight × (1− $\left(\frac{\mathrm{BF}\%}{\left(100\right)}\right)$ [35].

FM (If BF already known): Body Weight × $\left(\frac{\mathrm{BF}\%}{\left(100\right)}\right)$ [36].

FFMI: dividing Fat‐Free Mass (kg) by Height (m)^2^ [37].

FMI: dividing Fat Mass (kg) by Height (m)^2^ [36].

LAP in males: (WC [cm]−65) × (TG [mmol/L]) [38].

LAP in females: (WC [cm]−58) × (TG [mmol/L]) [38].

VAI in males: (WC/[39.68 + 1.88 × BMI]) × (TG [mmol/L]/1.03) × (1.31/HDL‐c [mmol/L]) [39].

VAI in females: (WC/[36.58 + 1.89 × BMI]) × (TG/0.81) × (1.52/HDL‐c) [39].

### 2.3 Statistical Analysis

The data analysis was performed using the Statistical Package for the Social Sciences (IBM SPSS statistics version 25). To determine statistically significant differences, independent‐samples *t*‐test and Mann–Whitney *U* test were used for normally and non‐normally distributed numerical variables, respectively, and chi‐square test was used for categorical variables. Descriptive statistics are presented as mean ± standard deviation (SD) for normally distributed continuous variables, median (interquartile range [IQR]) for non‐normally distributed continuous variables, and frequencies (percentages) for categorical variables. We modeled continuous PCE risk (%) with sex‐adjusted linear regression for individuals from 40 to 70 years old, entering one anthropometric index at a time. Simultaneous inclusion of multiple anthropometric indices within a single multivariable model was avoided due to the expected biological and statistical intercorrelation between these variables. Multicollinearity was assessed using tolerance and variance inflation factor (VIF) statistics, which demonstrated substantial multicollinearity among several anthropometric indices (Supporting Information S1: Table 1). Initial multivariable models including multiple anthropometric predictors yielded unstable and difficult‐to‐interpret coefficient estimates. To enable comparison across measures, all indices were standardized (*z*‐scores); regression coefficients therefore represent the change in risk per 1‐SD increment. We tested Index × Sex interactions in pooled models; when significant or clinically important, sex‐specific results are presented. For discrimination of elevated risk (≥7.5%) [19], we estimated AUCs (95% CIs) from ROC curves. Because greater lean mass is protective, FFM was inverted for pooled ROC so that larger values indicated higher risk; sex‐specific FFM ROC results are provided in the Supplement. For secondary analysis of patients below 40 years old, the binary high risk‐factor burden endpoint was employed to assess the discriminative impacts of indices. Besides, physical activity levels were estimated using the Metabolic Equivalent of Task (MET) values [40]. Vigorous activity was scored as 8 METs, moderate as 4 METs, and rest and sitting as 1 METs. Total weekly physical activity was calculated as MET‐min/week by multiplying MET value × min/day × days/week for each activity. Based on WHO and IPAQ criteria, participants were categorized as having low (<600 MET‐min/week), moderate (600−3000 MET‐min/week), or high (> 3000 MET‐min/week) physical activity levels [41, 42]. Likewise, to assess whether the 10‐year ASCVD risk score differed significantly across physical activity groups, a one‐way analysis of variance (ANOVA) was performed. A *p* value of less than 0.05 was considered statistically significant.

## 3 Results

The descriptive statistics of all the 1106 participants are shown in Table 1. It also presents a comparison of the baseline characteristics between gender groups. As indicated in Table 2, we analyzed 761 adults aged 40−70 years (444 men, 317 women). Median (IQR) of 10‐year ASCVD risk score was 1.33 (0.55−3.77) overall and was higher in men (2.67 [1.06−5.80]) than women (0.65 [0.36−1.45], *p* < 0.001). Figure 1 illustrates the distribution of 10‐year ASCVD risk score categories among study participants. Most participants were classified in the low‐risk category (73.1% overall), with a substantially higher proportion among women than men (92.1% vs. 59.5%, *p* < 0.001). The borderline, intermediate, and high‐risk categories accounted for 11.6%, 12.9%, and 2.5% of participants, respectively. Among men, the corresponding proportions were 16.4%, 20.3%, and 3.8%, compared with 4.7%, 2.5%, and 0.6% among women. Men were older and had higher systolic and diastolic blood pressure; women had higher HDL‐C. LDL‐C did not differ by sex (119.37 ± 32.19 vs. 121.24 ± 30.48 mg/dL, *p* = 0.417). Total cholesterol was higher in women (199.13 ± 41.58 vs. 191.79 ± 41.88 mg/dL, *p* = 0.017), whereas TG were higher in men (159.54 ± 82.85 vs. 135.20 ± 67.22 mg/dL, *p* < 0.001). Current smoking was more frequent in men (18.0% vs. 6.9%, *p* < 0.001); diabetes prevalence was similar (10.8% vs. 8.8%, *p* = 0.37). Table 1 in the supplemental materials displays the anthropometric indices of the participants categorized by gender and age.

**Table 1:** Baseline characteristics of all subjects by gender groups.

Characteristic	All participants	Male (%)	Female (%)	*p* value^a^
No. (*N*)	1106	605 (54.70)	501 (45.30)	—
Age (Year)	44.34 ± 8.86	45.60 ± 9.18	42.83 ± 8.20	< 0.001
Systolic BP (mm Hg)	118.47 ± 15.34	121.88 ± 14.89	114.35 ± 14.87	< 0.001
Diastolic BP (mm Hg)	78.16 ± 10.24	80.45 ± 9.86	75.38 ± 10.01	< 0.001
HDL‐C (mg/dL)	46.46 ± 10.16	43.65 ± 9.11	49.85 ± 10.33	< 0.001
LDL‐C (mg/dL)	117.27 ± 30.84	118.55 ± 31.11	115.72 ± 30.47	0.128
TC (mg/dL)	190.63 ± 40.64	190.29 ± 40.76	191.03 ± 40.53	0.762
TG (mg/dL)	142.44 ± 74.46	156.02 ± 80.82	126.03 ± 62.21	< 0.001
Current smoking status (*N*)	132 (11.90%)	107 (17.70%)	25 (5.00%)	< 0.001
Diabetes status (*N*)	96 (8.70%)	52 (8.60%)	44 (8.80%)	0.912
*Educational attainment (N)*				0.082
Less than high school	104 (9.40%)	48 (7.90%)	56 (11.2%)	
High school degree	255 (23.1%)	133 (22%)	122 (24.4%)	
At least some college	747 (67.5%)	424 (70.1%)	323 (64.5%)	

*Note:* Continuous variables are presented as mean ± SD or median (IQR), as appropriate, while categorical variables are presented as frequencies and percentages.

Abbreviations: BP, blood pressure; HDL‐C, high‐density lipoprotein cholesterol; LDL‐C, low‐density lipoprotein cholesterol; TC, total cholesterol; TG, triglyceride.

^a^ Statistical significance was considered < 0.05.

**Table 2:** Baseline Risk factors of 10‐year risk of a first atherosclerotic cardiovascular disease (ASCVD) event by gender groups for patients between age 40 and 70.

Characteristic	All participants	Male (%)	Female (%)	*p* value^a^^,^ b
No. (*N*)	761	444 (58.3%)	317 (41.7%)	—
10‐year ASCVD risk score^b^	1.33 (0.55−3.77)	2.67 (1.06−5.80)	0.65 (0.36−1.45)	< 0.001
Low‐risk: < 5%	556 (73.1%)	264 (59.5%)	292 (92.1%)	< 0.001
Borderline‐risk: 5%−7.49%	88 (11.6%)	73 (16.4%)	15 (4.7%)	
Intermediate‐risk: 7.5%−20%	98 (12.9%)	90 (20.3%)	8 (2.5%)	
High‐risk: ≥ 20%	19 (2.5%)	17 (3.8%)	2 (0.6%)	
Age (Year)	48.50 ± 7.04	49.23 ± 7.31	47.49 ± 6.51	< 0.001
Systolic BP (mm Hg)	120.71 ± 15.44	122.84 ± 14.54	117.71 ± 16.16	< 0.001
Diastolic BP (mm Hg)	79.42 ± 10.17	81.2 ± 9.39	76.92 ± 10.70	< 0.001
HDL‐C (mg/dL)	46.76 ± 10.34	44.06 ± 9.43	50.55 ± 10.39	< 0.001
LDL‐C (mg/dL)	120.15 ± 31.48	119.37 ± 32.19	121.24 ± 30.48	0.417
TC (mg/dL)	194.85 ± 41.88	191.79 ± 41.88	199.13 ± 41.58	0.017
TG (mg/dL)	149.40 ± 77.61	159.54 ± 82.85	135.20 ± 67.22	< 0.001
Current smoking status (*N*)	102 (13.4%)	80 (18%)	22 (6.9%)	< 0.001
Diabetes status (*N*)	76 (10%)	48 (10.8%)	28 (8.8%)	0.37

*Note:* Quantitative variables are presented as mean ± standard deviation, while categorical variables are presented as frequencies and percentages.

Abbreviations: ASCVD, atherosclerotic cardiovascular disease risk score; BP, blood pressure; HDL‐C, high‐density lipoprotein cholesterol; LDL‐C, low‐density lipoprotein cholesterol; TC, total cholesterol; TG, triglyceride.

^a^ Statistical significance was considered < 0.05;

^b^ It was estimated using the ASCVD risk score estimator as per the 2019 ACC/AHA guideline. Gender, age, race, cholesterol profile, blood pressure, use of antihypertensive therapy, DM, and smoking status were required to calculate the risk.

**Figure 1:**
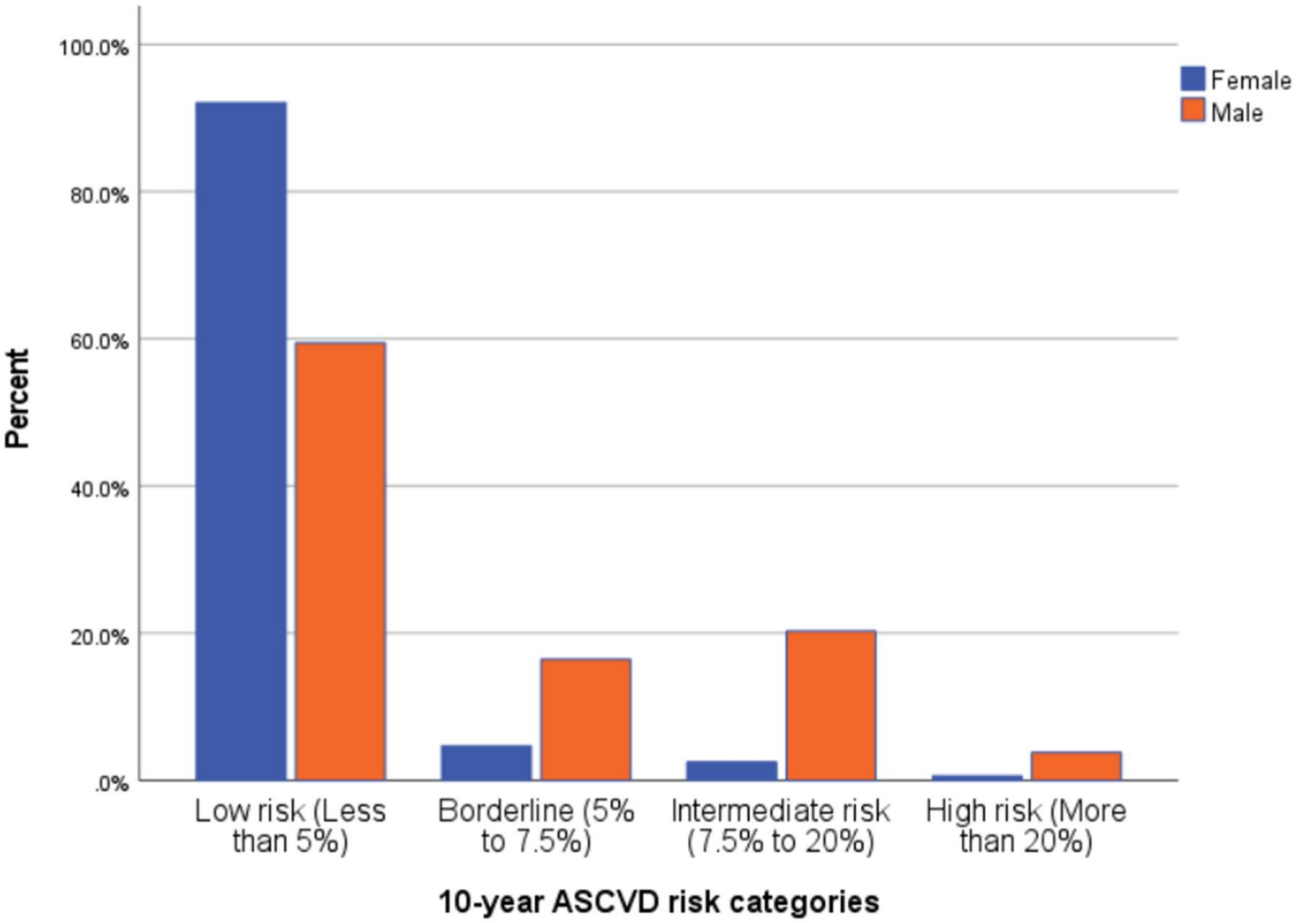
Distribution of 10‐year ASCVD risk categories by sex.

### 3.1 Association of Anthropometric Indices With Continuous ASCVD Risk (Age 40−70 Years)

In sex‐adjusted linear models, higher WHtR, WHR, BF, WC, FMI, LAP, and VAI were each associated with higher ASCVD risk (all *p* < 0.001). Effect sizes were B = 0.236 for WHtR (95% CI 0.168−0.304), 0.220 for WHR (0.152−0.288), 0.272 for BF (0.207−0.336), 0.162 for WC (0.096−0.228), 0.197 for FMI (0.131−0.263), 0.196 for LAP (0.130–0.261), and 0.171 for VAI (0.105−0.237) (all *p* < 0.001). FFM was inversely related to risk (*B* = −0.176, 95% CI −0.257 to −0.095; *p* < 0.001). HC was not associated (*B* = 0.051; *p* = 0.138) and FFMI was null (*B* = −0.003; *p* = 0.927). Table 3 shows sex‐adjusted linear associations. Figure 2 illustrates the relationship between ASCVD risk score and selected notable anthropometric and fat distribution indices using scatter plots stratified by sex.

**Table 3:** Association of different anthropometric and metabolic indices with the 10‐year risk of a first ASCVD event using linear regression model (sex‐adjusted) in participants from 40 to 70 years old.

	All cases			
	Unstandardized Coefficients B	95% CI	Adjusted *R* ^2^	*p* value
WC (Cm)	0.162	[0.096; 0.228]	0.287	< 0.001
WHR (unitless (ratio))	0.220	[0.152; 0.288]	0.302	< 0.001
WHtR (unitless (ratio))	0.236	[0.168; 0.304]	0.307	< 0.001
HC (cm)	0.051	[−0.016; 0.118]	0.267	0.138
BMI (Kg/m^2^)	0.146	[0.079; 0.212]	0.283	< 0.001
BAI (%)	0.221	[0.134; 0.308]	0.289	< 0.001
BF(%)	0.272	[0.207; 0.336]	0.326	< 0.001
FM (Kg)	0.141	[0.074; 0.208]	0.281	< 0.001
FFM (Kg)	−0.176	[−0.257; −0.095]	0.282	< 0.001
FMI (Kg/m^2^)	0.197	[0.131; 0.263]	0.297	< 0.001
FFMI (Kg/m^2^)	−0.003	[−0.071; 0.064]	0.265	0.927
LAP (Index score)	0.196	[0.130; 0.261]	0.297	< 0.001
VAI (Index score)	0.171	[0.105; 0.237]	0.289	< 0.001

Abbreviations: BAI, body adiposity index; BF, body fat; BMI, body mass index; FFM: fat‐free mass; FFMI, fat‐free mass index; FM, fat mass; FMI, fat mass index; HC, hip circumference, LAP, lipid accumulation product; VAI, visceral adiposity index; WC, waist circumference, WHR, waist‐to‐hip ratio, WHtR, waist‐to‐height ratio.

**Figure 2:**
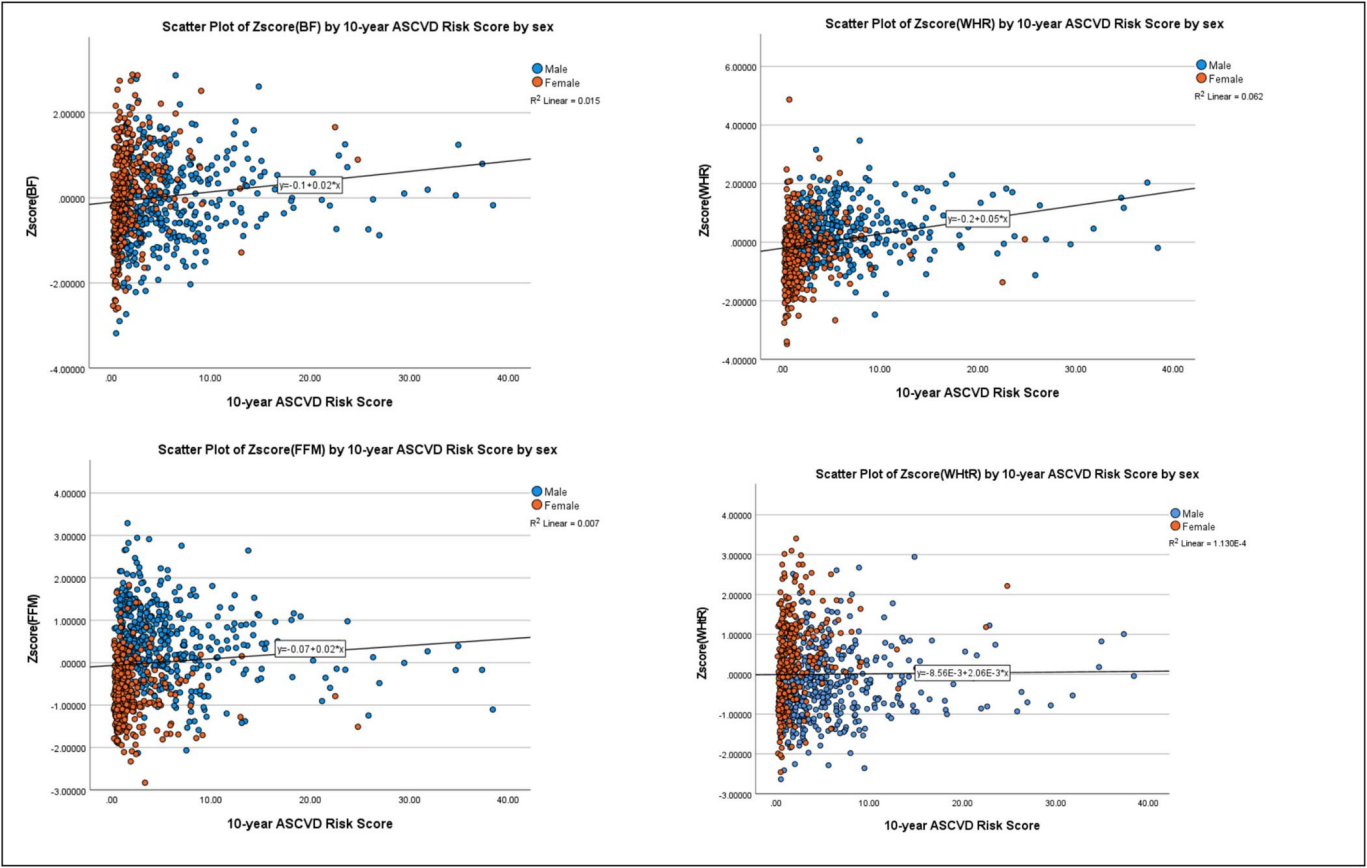
Notable anthropometric and fat distribution indices based on 10‐year ASCVD Risk Score.

### 3.2 Sex‐Specific Analyses and Tests for Interaction (Age 40−70 Years)

Sex‐specific models showed broadly similar positive associations of BF and WHtR with risk in both men and women, and interaction tests were non‐significant for these indices (interaction *p* = 0.501 and 0.479, respectively). In contrast, several indices demonstrated sex modification. WHR showed a larger slope in men than women (interaction *p* = 0.046); BMI (interaction *p* = 0.044) and FFMI (interaction *p* = 0.007) also differed by sex. Metabolic indices incorporating lipids (LAP, VAI) showed strong interactions (both *p* ≤ 0.001). FFM was strongly negative in men (*B* = −0.253, 95% CI −0.355 to −0.152; *p* < 0.001) and essentially null in women (*B* = −0.013; *p* = 0.848). Full coefficients and 95% CIs are provided in Table 4.

**Table 4:** Association of different anthropometric and metabolic indices with the 10‐year risk of a first ASCVD event using linear regression model (sex‐adjusted) in participants between 40 and 70 years old by gender.

	Male (*N* = 444)			Female (*N* = 317)			*p* value for interaction
	Unstandardized coefficients *B*	95% CI	*p* value	Unstandardized coefficients *B*	95% CI	*p* value	
WC (Cm)	0.107	[0.012; 0.212]	0.027	0.225	[0.137; 0.314]	< 0.001	0.079
WHR (unitless [ratio])	0.293	[0.191; 0.396]	< 0.001	0.155	[0.067; 0.243]	0.001	0.046
WHtR (unitless [ratio])	0.211	[0.107; 0.314]	< 0.001	0.260	[0.173; 0.347]	< 0.001	0.479
HC (cm)	−0.065	[−0.172; 0.042]	0.231	0.141	[0.058; 0.223]	< 0.001	0.003
BMI (Kg/m^2^)	0.078	[−0.021; 0.177]	0.123	0.215	[0.128; 0.301]	< 0.001	0.044
BAI (%)	0.173	[0.030; 0.316]	0.018	0.254	[0.151; 0.358]	< 0.001	0.365
BF(%)	0.250	[0.154; 0.346]	< 0.001	0.294	[0.211; 0.377]	< 0.001	0.501
FM (Kg)	0.078	[−0.015; 0.172]	0.099	0.227	[0.133; 0.321]	< 0.001	0.031
FFM (Kg)	−0.253	[−0.355; −0.152]	< 0.001	−0.013	[−0.149; 0.123]	0.848	0.007
FMI (Kg/m^2^)	0.151	[0.052; 0.251]	0.003	0.241	[0.157; 0.325]	< 0.001	0.182
FFMI (Kg/m^2^)	−0.113	[−0.209; −0.018]	0.020	0.128	[0.035; 0.220]	0.007	< 0.001
LAP (Index score)	0.087	[−0.003; 0.177]	0.057	0.359	[0.269; 0.450]	< 0.001	< 0.001
VAI (Index score)	0.080	[−0.012; 0.172]	0.090	0.294	[0.204; 0.385]	< 0.001	0.001

*Note: p* value for interaction formally tests whether male and female slopes differ.

Abbreviations: BAI, body adiposity index; BF, body fat; BMI, body mass index; FFM: fat‐free mass; FFMI, fat‐free mass index; FM, fat mass; FMI, fat mass index; HC, hip circumference, LAP, lipid accumulation product; VAI, visceral adiposity index; WC, waist circumference, WHR, waist‐to‐hip ratio, WHtR, waist‐to‐height ratio.

### 3.3 Discrimination of Elevated ASCVD Risk (≥ 7.5%; Age 40−70 Years)

In ROC analyses, WHR provided the best discrimination (AUC 0.646, 95% CI 0.594−0.698; *p* < 0.001). BF showed modest discrimination (AUC 0.556, 0.503−0.608; *p* = 0.055) and WC was borderline (AUC 0.543, 0.489−0.598; *p* = 0.138), while WHtR (AUC 0.489) and BMI (AUC 0.477) were not different from chance (Table 5 and Figure 3, ROC curve analysis for all anthropometric indices is presented in Supporting Information S1: Table 3). Sex‐specific ROC curves demonstrated that in men, FFM (inverse orientation) and WHR discriminated best (AUC 0.649 and 0.606, respectively), whereas BF and WHtR were modest (AUC 0.591 and 0.566) (Supporting Information S1: Table 4). In women, AUC estimates were imprecise given the small number of elevated‐risk cases; the highest point estimates were for WHtR (AUC 0.644) and WC (0.625), with wide confidence intervals.

**Table 5:** Diagnostic performance (AUC, sensitivity, specificity, and cut‐off values) of anthropometric indices for identifying elevated 10‐year ASCVD risk (7.5% threshold) in adults aged 40‐70 years by ROC curve analysis.

Anthropometric index	AUC	95% CI	*p* value	Optimal cut‐off	Sensitivity	Specificity	Youden index
WHR (unitless [ratio])	0.646	[0.594; 0.698]	< 0.001	0.905*	0.867	0.345	0.211
BF(%)	0.556	[0.503; 0.608]	0.055	38.174	0.658	0.466	0.124
WC (Cm)	0.543	[0.489; 0.598]	0.138	89.900	0.800	0.281	0.081
WHtR (unitless [ratio])	0.489	[0.435; 0.543]	0.717	0.511	0.917	0.144	0.061
BMI (Kg/m^2^)	0.477	[0.422;0.533]	0.430	23.887	0.825	0.199	0.024

Abbreviations: BF, body fat; BMI, body mass index; WC, waist circumference; WHR, waist‐to‐hip ratio; WHtR, waist‐to‐height ratio.

* Although the cutoff value of 0.936 demonstrated a slightly higher Youden index (Youden index = 0.213; sensitivity = 63.3%, specificity = 57.9%), the secondary cutoff point was ultimately selected due to its higher sensitivity, which was considered more important for cardiovascular risk screening and primary prevention.

**Figure 3:**
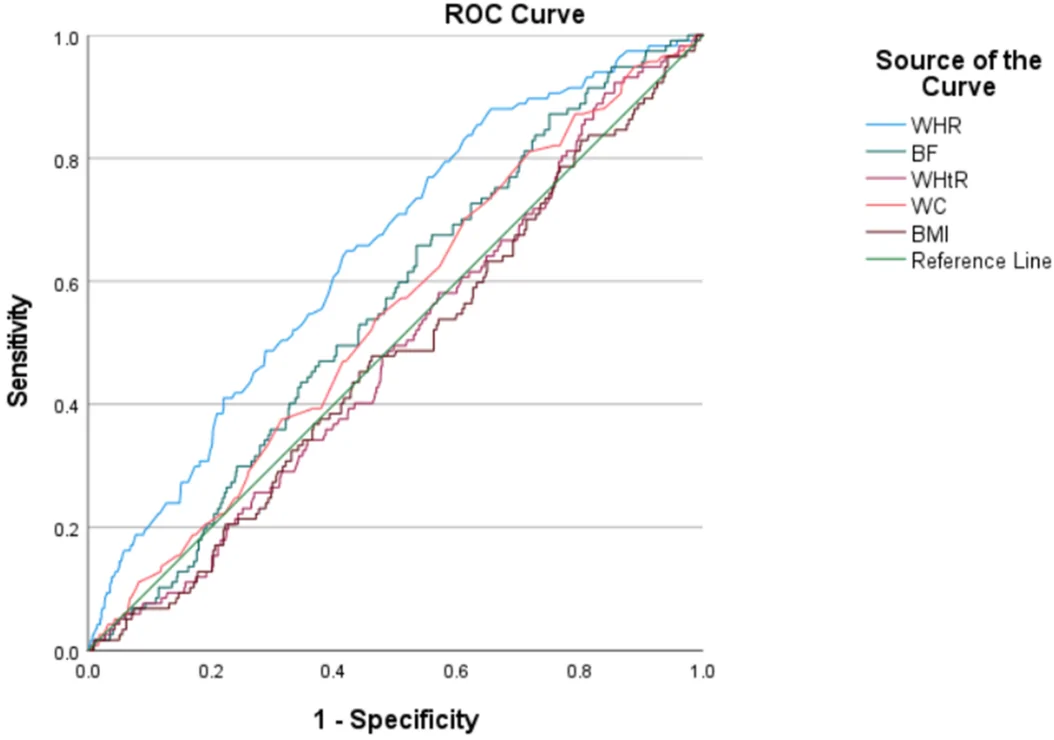
pooled ROC for the 40–70 group.

### 3.4 Secondary Analysis: Participants ≪ 40 Years

Because 10‐year PCE risk is near‐zero and not validated in younger adults, we evaluated discrimination for a composite high risk‐factor burden endpoint. Indices capturing adiposity discriminated well: WC (AUC 0.747, 95% CI 0.695−0.798), BF (0.728, 0.674−0.782), FMI (0.727, 0.673−0.781) and BMI (0.726, 0.672−0.780). WHR and WHtR also performed well (AUC 0.682 and 0.681, respectively). Figure 4 and Supporting Information S1: Table 5 show the secondary analysis of individuals less than 40 years old.

**Figure 4:**
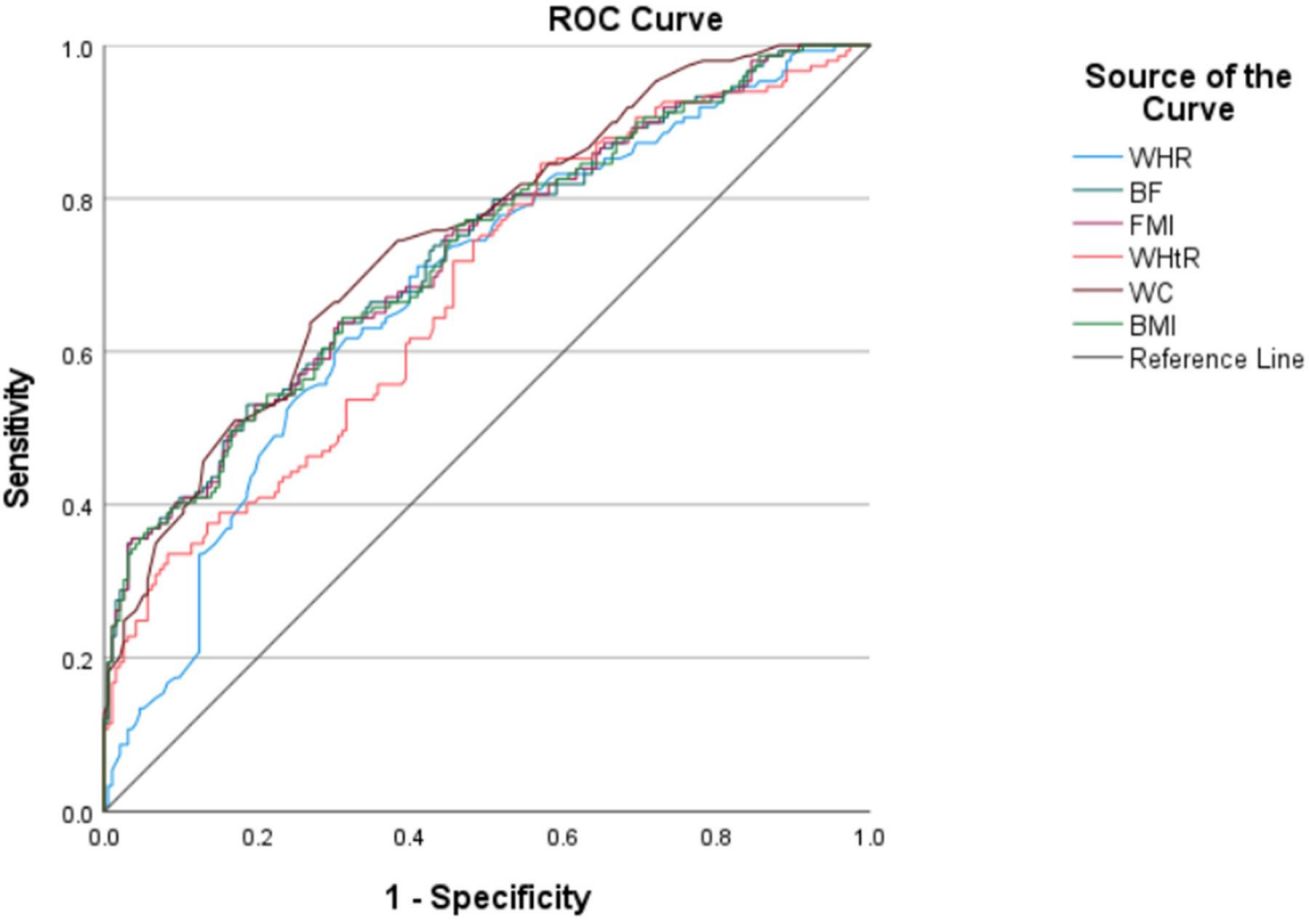
Secondary analysis on individuals below 40 years old.

Based on total MET‐min/week, 19.4% of participants were categorized as low, 52.1% as moderate, and 28.5% as high physical activity level (Supporting Information S1: Table 6). Besides, the one‐way ANOVA indicated that there was no statistically significant difference in mean ASCVD risk score among the low, moderate, and high physical activity groups (*F*(2, 1082) = 1.202, *p* = 0.301).

## 4 Discussion

In this cross‐sectional analysis of 761 adults aged 40–70 years, the mean 10‐year ASCVD risk score was relatively low (4.15), with men showing significantly higher scores than women (5.75 vs. 1.92, *p* < 0.001). In our linear regression models, BF showed the strongest positive association with ASCVD risk (*B* = 0.272) (Figure 2), followed by WHtR (*B* = 0.236), WHR (*B* = 0.220), and WC (*B* = 0.162). In contrast, FFM was inversely associated with ASCVD risk. Sex‐stratified analyses revealed distinct patterns. Among female participants, FFMI was positively associated with ASCVD risk (*B* = 0.128, *p* = 0.007), whereas in men, FFM exhibited a strong inverse association (*B* = −0.253, *p* < 0.001). Additionally, several other adiposity indices, including FMI and BF, were positively associated with ASCVD risk in women, while FFMI and FFM showed stronger correlations in men, highlighting the importance of considering sex‐specific body composition in cardiovascular risk assessment.

Obesity is a well‐established contributor to cardiometabolic disorders [43, 44]. While several studies have examined the association between obesity and cardiovascular risk [11, 20, 45, 46, 47], research evaluating detailed fat distribution indices alongside traditional anthropometric measures in relation to ASCVD risk remains limited.

Our findings demonstrated significant associations of central adiposity measures, particularly WHR and WC, with estimated ASCVD risk. Similar to our results, Wu et al. reported WHR as the strongest predictor in men (AUC = 0.71), and WHtR in women (AUC = 0.75). Conversely, A Body Shape Index (ABSI) showed the lowest predictive ability in that study [11]. On the other hand, analyses of the NHANES dataset found ABSI to be highly predictive [20], highlighting potential population‐specific differences.

Comparisons with international evidence further align with our results. Findings from an American cross‐sectional study consisting mostly of overweight and obese subjects showed a linear increase of BMI, weight, and WHtR with cardiovascular risk among diabetic and pre‐diabetic patients [48]. In comparison, our study population included a larger proportion of individuals with normal BMI. Similarly, a European prospective study with a median follow‐up of 7.9 years demonstrated that all anthropometric measurements, including BMI, WC, WHR, and WHtR, increased the risk of CVD mortality [49], whose anthropometric profiles were more comparable to our female participants than to our male participants. Supporting prior evidence, we observed that each unit increase in WHR and WC corresponded to a 0.240 and 0.114 increase in ASCVD risk score, respectively.

Consistent with our findings, observational studies across different ethnicities reveal positive associations between WC and CVD risk [50, 51, 52, 53]. A systematic review and meta‐analysis in the South Asian population, further confirmed that higher WC and WHR were associated with CVD incidents [54]. However, data from the NHIS cohort in an Asian population indicated no significant impact of WC or WHR on CVD morbidity and mortality [55]. In our sex‐stratified analyses, higher correlations were observed among female participants compared to males. Previous studies have also suggested that females may experience higher CVD risk at higher WC measurements [50, 56, 57], this could be explained by the fact that female typically have greater BF percentages than males at comparable WC values [58].

Regardless of sex, we observed a positive correlation between WHR and ASCVD risk. WHR, a well‐recognized anthropometric index for central obesity [59], has been consistently shown to predict CVD morbidity and mortality [60, 61]. In line with our results, Darbandi et al. reported that WHR provide superior predictive ability for CVD events [62]. Of note, Higher WHR has been associated with reduced lower‐limb muscle mass and adverse metabolic profiles in prior studies [63, 64].

In the current study, we revealed that WHtR and BF were significantly correlated to ASCVD risk score. WHtR has been recognized as a strong predictor of CVD risk and mortality. A recent systematic review and meta‐analysis reported that categorical and continuous measurements of WHtR increased CVD mortality risk by 39% and 19%, respectively [65]. A prospective population‐based study conducted in MENA region also found strong association between WHtR and CVD mortality in individuals under 65 [66]. Using data from the NHANES cohort consisting of pre‐diabetic and diabetic individuals, He et al. claimed that participants with higher WHtR were more likely to be at risk of CVD outcomes [48].

In contrast to our results, Bazadona et al. reported no significant correlation between BF and cIMT, a well‐known indicator of atherosclerosis, in an obese population with a mean BMI of 43. 47 ± 9.4 kg/m^2^ [67]. However, other evidence suggests that BF distribution may be a better predictor of coronary artery disease than BMI alone in obese individuals [68]. As it has been interpreted from previous studies, inflammation and oxidative stress caused by obesity can lead to vascular abnormalities related to atherosclerosis and CVD events [69, 70]. The discrepancy with our findings may be explained by differences in study populations, as our cohort primarily included non‐obese adults, whereas Bazadona et al. investigated individuals with severe obesity.

Our results also highlight the role of FM and related indices. Women with higher FM, FMI, and FFMI were more likely to exhibit elevated ASCVD risk, while in men, higher FFM and FFMI showed protective associations. One possible explanation for these findings is the close relationship between FFM and FM. Previous studies have demonstrated that increases in FM may lead to adipose tissue infiltration within skeletal muscle and other organs such as the liver and pancreas, thereby contributing to an apparent increase in measured FFM [71]. In our study, females demonstrated higher levels of FM across the overall population and within different age groups compared with males. This may suggest that higher FFM or FFMI values in women could partly reflect greater adiposity, ectopic fat deposition, insulin resistance, and a more sedentary lifestyle rather than metabolically protective lean tissue alone. These factors may subsequently contribute to higher estimated ASCVD risk scores observed in women [71, 72]. Using a Mendelian randomization design, Larsson et al. suggested causal association between FMI and CVD events, whereas FFMI shows limited genetically predicted association with most CVD outcomes [73]. These findings confirm the importance of evaluating both fat and lean mass distribution, rather than relying solely on BMI.

BMI provides a general estimate of obesity with considerable limitations in CVD risk evaluation. BMI may misclassify individuals with high muscle mass as overweight/obese or fail to identify individuals with normal BMI but high fat accumulation, limiting its sensitivity for cardiovascular risk detection [74, 75, 76]. More importantly, BMI provides no information about fat distribution. This distinction is particularly relevant because adipose tissue is not metabolically homogeneous: visceral adipose tissue is more strongly associated with insulin resistance, dyslipidemia, hypertension, and chronic low‐grade inflammation than subcutaneous adipose tissue [74]. Visceral adiposity may also be accompanied by ectopic fat deposition in metabolically relevant organs and tissues, including the liver and skeletal muscle, which may further contribute to adverse metabolic and cardiovascular profiles. Thus, individuals with similar BMI values may have substantially different distributions of adipose tissue and, consequently, different cardiovascular risk profiles.

Central and visceral adiposity may confer greater cardiometabolic risk than predominantly subcutaneous fat because visceral adipose tissue is metabolically active and associated with insulin resistance, dyslipidemia, hypertension, and chronic low‐grade inflammation. These mechanisms provide a potential biological explanation for why waist‐based measures, which capture abdominal body size and indirectly reflect central adiposity, may provide information about cardiovascular risk that is not captured by BMI alone.

Previous studies have similarly reported that WHR and WHtR may provide stronger associations with CVD risk than BMI, potentially reflecting their ability to capture aspects of BF distribution that BMI does not capture [77, 78, 79]. These biological differences may partly explain why waist‐based measures, particularly WHR in our study, provided additional information beyond BMI. WHR showed the highest discrimination for elevated 10‐year ASCVD risk (AUC 0.646), whereas BMI showed no meaningful discriminatory ability (AUC 0.477). In the linear regression analysis, WHR, WHtR, and WC were positively associated with continuous ASCVD risk, while BMI was not significant. As a result, these indices can potentially provide more nuanced results in ASCVD risk prediction than traditional anthropometric indices. However, some of the studies found BRI as the strongest predictor of CVD risk, which can be attributed to their different population target to our study [80, 81]. Although, WHR and WHtR remained as significant contributors to CVD risk in their study, more than traditional factors like BMI. Similar to our findings, Sadeghi et al. found ABSI and BAI to have lower AUC than WHR and WHtR in both genders. They also found ABSI to be a stronger predictor than BMI, considering its higher specificity and AUC, though BMI showed higher sensitivity [10].

Finally, the current study adds to growing evidence of gender‐specific associations of anthropometric indices with CVD outcomes. Regional studies in Saudi Arabia and Iran have reported higher CVD risk among women with elevated WHR compared to men of similar age and risk profiles [82, 83], whereas in other populations, men with increased WC may be at greater risk of severe CAD events [84]. Recent consensus recommendations emphasize that women and men may differ in cardiovascular risk profiles, underlying pathophysiology, and responses to preventive strategies, supporting more individualized approaches to cardiovascular risk assessment [85, 86]. These differences may partly reflect sex‐related variation in adipose tissue distribution and metabolism. Compared with men, women generally have a greater proportion of subcutaneous adipose tissue, whereas men tend to accumulate more visceral adipose tissue; hormonal changes, particularly those accompanying menopause, may further promote visceral fat accumulation and adverse metabolic changes in women [87, 88]. These biological differences may also contribute to sex‐specific associations between body composition measures and cardiovascular risk. In our study, sex differences were particularly evident for WHR, BMI, FFM, FFMI, LAP, and VAI, for which the interaction between sex and the respective index was statistically significant. Notably, FFM was inversely associated with ASCVD risk in men (*B* = −0.253, *p* < 0.001), whereas its association was not significant in women (*B* = −0.013, *p* = 0.848). Similarly, FFMI showed an inverse association in men but a positive association in women (*B* = −0.113 vs. 0.128; *p* for interaction < 0.001). These findings suggest that the relationship between body composition and cardiovascular risk may differ by sex, supporting the value of sex‐stratified analyses. The divergent associations observed for FFM and FFMI may partly relate to sex‐specific differences in body composition, fat distribution, and metabolic physiology.

Notably, our study population consisted of adults with a mean age of 44.3 years, which differs considerably from adolescent populations. In younger cohorts, such as 14‐year‐old Australians, anthropometric measurements may not yet strongly predict CVD risk, reflecting how CVD risk factors evolve with age [89].

Simple anthropometric measures can help identify unfavorable adiposity patterns and cardiometabolic risk before overt cardiovascular disease develops, while requiring minimal resources. In particular, WHR and WC can be obtained easily in routine clinical settings without laboratory testing or imaging, making them practical tools for large‐scale screening and repeated risk assessment. Although our study focused on adults, evidence from younger populations also supports the role of anthropometric assessment as part of early cardiovascular prevention and lifestyle‐oriented risk reduction across the life course [12]. Their simplicity and low cost may enhance their feasibility in primary prevention settings, particularly where access to laboratory or imaging‐based assessment is limited.

One of the main strengths of this study is the use of a large, population‐based sample, which increases confidence in our findings. Another important strength is that we examined sex‐specific associations across a wide range of anthropometric and FM–related measures. This approach provides new insights into how body composition differs between men and women and how these differences relate to the 10‐year PCE‐based ASCVD risk. Very few studies have explored such a broad set of measures in a relatively non‐obese Middle Eastern population.

Our results revealed clear differences between sexes. In men, fat‐free mass (FFM) was linked to lower ASCVD risk, while in women, higher FM–related indices, including FFMI, were associated with higher risk. This highlights the value of considering sex‐specific body composition when assessing cardiovascular risk. Clinically, measures like WC, WHR, and FFMI could help identify individuals at higher risk, especially women, and could guide public health strategies aimed at reducing central obesity.

There are some limitations to consider. First, this was a cross‐sectional study conducted at a single center, and therefore the findings should be interpreted in the context of the characteristics of this specific cohort. Our population was fairly homogeneous in terms of ethnicity, occupation, and possibly socioeconomic status, which may limit how broadly the findings apply. Due to the lack of sufficient longitudinal follow‐up and the absence of adjudicated cardiovascular event data, it was not possible to assess actual cardiovascular events or establish temporal or causal relationships. Therefore, the 10‐year ASCVD risk score was used as the primary outcome of interest as a surrogate estimate of cardiovascular risk. Consequently, the observed predictive performance of the anthropometric indices should be interpreted with caution. Measurement error is another potential issue: small differences in waist or hip measurements can affect indices like WHR and WHtR. To reduce this risk, all measurements were taken by trained clinicians using standardized procedures. Another limitation of the present study is that fat and lean body mass were estimated using anthropometric predictive equations rather than direct or instrument‐based body‐composition assessment methods such as dual‐energy X‐ray absorptiometry (DXA) or bioelectrical impedance analysis. Although these methods may provide more precise estimates of body composition, the present study primarily aimed to evaluate simple, low‐cost, and widely accessible anthropometric measures applicable to routine clinical practice and large‐scale primary prevention settings. Finally, the cross‐sectional design also limits causal inference and prevents determination of the directionality of the observed associations, emphasizing the need for longitudinal studies to clarify the direction and mechanisms underlying these associations.

## 5 Conclusions

In this population‐based study, central adiposity and body composition indices, particularly WHR and sex‐specific FFM measures, were associated with estimated 10‐year ASCVD risk. These findings support the potential value of incorporating BF distribution measures into cardiovascular risk assessment, while highlighting important sex‐specific differences. In particular, significant sex‐by‐index interactions were observed for WHR, BMI, FFM, FFMI, LAP, and VAI, underscoring the importance of considering sex when interpreting body composition and adiposity measures. Our sex‐stratified analysis revealed that these associations were more pronounced in women, highlighting the need for targeted primary prevention in this group. Although WHR and WC are established markers of central obesity, our findings suggest that FFMI, a relatively under‐studied index, demonstrated sex‐specific associations with ASCVD risk, particularly among women, whereas FFM showed an inverse association in men.

These observations emphasize the value of considering sex‐specific body composition when assessing CVD risk. Beyond risk prediction, WHR and WC are simple, low‐cost measures that require no laboratory testing or imaging and may therefore be readily incorporated into routine preventive cardiovascular assessment, particularly for early identification of unfavorable adiposity patterns. Future studies should explore how genetic and environmental factors influence these anthropometric measures, which could help design more effective, personalized strategies for preventing ASCVD.

## Author Contributions

**Mohammad Javad Zibaeenezhad:** conceived the idea for the manuscript. Data providing. **Alireza Arzhangzadeh:** data providing. Writing and making the revisions. **Mehrab Sayadi:** data providing. **Alireza Khodadadiyan:** statisitical analysis, manuscript writing and making revisions. **Melika Golmohammadi:** statistical analysis, manuscript writing and making revisions. **Zahra Hooshanginezhad:** manuscript writing. **Ebrahim Rahimi:** statisitical analysis, manuscript writing and making revisions. **Maryam Jafari:** manuscript writing and making revisions. **Mehdi Bazrafshan:** manuscript writing and making revisions. **Nader Parsa:** data providing. **Iman Razeghian‐Jahromi:** data providing and making revisions. **Roozbeh Narimani‐Javid:** manuscript writing and making revisions. **Seyyed Saeed Mohammadi:** manuscript writing and making revisions. **Fatemeh Zibaeenejad:** data providing, conceived the idea for the manuscript. **Davar Aldavood:** manuscript writing and making the revisions. **Hamed Bazrafshan Drissi:** supervised the study, revising the manuscript, and making the revisions. Approved the final version of the manuscript. **Masih Jamshidi:** making the revisions.

## Funding

The authors have nothing to report.

## Ethics Statement

The ethical number of our study is IR.SUMS.MED.REC.1401.568. The study was conducted based on the principles of the Declaration of Helsinki and the approval of the ethics committee of Shiraz University of Medical Sciences.

## Consent

All participants involved in this study have provided informed consent for the publication of their children's information and data.

## Conflicts of Interest

The authors declare no conflicts of interest.

## Supporting information


Supporting File

## Data Availability

The data that support the findings of this study are available from the corresponding author upon reasonable request. The related materials and analysed data can be requested from the authors. Kindly inform the corresponding author if you are willing in such data.
