# Supplementary material for: Which Anthropometric Index Best Signals 10‑Year Cardiovascular Risk? A Sex‑Stratified Analysis From the Shiraz Heart Study
Source: Clin Cardiol. 2026 Nov 9;49(11):e70524. doi: 10.1002/clc.70524 (PMC13748821; doi:10.1002/clc.70524)
Supplement: Supplementary file 1 [file CLC-49-e70524-s001.docx]

Table S1: Variance Inflation Factor (VIF) and Tolerance Statistics for Assessment of Multicollinearity Among Anthropometric Indices

| **Variable** | **Tolerance** | **VIF** | **Interpretation** |
| --- | --- | --- | --- |
| HC | 0.002 | 486.747 | Severe multicollinearity |
| WC | 0.001 | 1880.561 | Severe multicollinearity |
| WHR | 0.005 | 197.226 | Severe multicollinearity |
| BAI | 0.002 | 503.792 | Severe multicollinearity |
| WHtR | 0.001 | 1545.509 | Severe multicollinearity |
| BF | 0.035 | 28.324 | Severe multicollinearity |
| FM | 0.005 | 205.965 | Severe multicollinearity |
| FMI | 0.005 | 189.150 | Severe multicollinearity |
| FFM | 0.007 | 142.904 | Severe multicollinearity |
| FFMI | 0.021 | 46.824 | Severe multicollinearity |

| Table S2: Anthropometric indices of all subjects study categorized by gender and age | | | | | |
| --- | --- | --- | --- | --- | --- |
| **Age** |  | **26-72y** | **26-44.9y** | **45-59.9y** | **≥60y** |
| **Male** | **n** | **605** | **308** | **247** | **50** |
| Height | cm | 172.72±7.19 | 174.77±6.71 | 171.07±7.04 | 168.21±6.80 |
| Weight | kg | 79.60±12.41 | 81.12±12.69 | 78.97±11.87 | 73.31±11.08 |
| WC | cm | 96.06±9.04 | 95.77±9.13 | 96.69±8.84 | 94.77±9.46 |
| WHR | % | 94.22±4.89 | 93.18±4.36 | 95.21±5.03 | 95.66±5.80 |
| WHtR | % | 55.68±5.42 | 54.84±5.26 | 56.59±5.39 | 56.39±5.80 |
| BMI | kg/m^2^ | 26.67±3.77 | 25.37±3.54 | 26.98±3.77 | 25.88±3.48 |
| BAI | % | 26.97±3.46 | 26.51±3.32 | 27.46±3.58 | 27.45±3.45 |
| BF | % | 26.29±4.97 | 24.43±6.68 | 27.93±4.55 | 29.60±4.15 |
| FM | kg | 20.54±6.91 | 20.82±7.12 | 20.67±6.79 | 18.15±5.82 |
| FFM | kg | 59.06±6.21 | 60.31±6.21 | 58.30±5.84 | 55.16±5.84 |
| FMI | kg/m^2^ | 6.89±2.31 | 6.82±2.32 | 7.08±2.33 | 6.42±2.05 |
| FFMI | kg/m^2^ | 19.78±1.47 | 19.72±1.49 | 19.91±1.44 | 19.47±1.43 |
| **Female** | **n** | **501** | **316** | **164** | **21** |
| Height | cm | 158.97±5.96 | 160.07±5.61 | 157.43±6.06 | 154.32±5.83 |
| Weight | kg | 68.76±11.88 | 67.99±12.07 | 70.41±11.56 | 67.46±10.48 |
| WC | cm | 93.47±10.01 | 92.06±9.78 | 95.89±9.99 | 95.79±9.81 |
| WHR | % | 90.39±6.38 | 89.67±6.61 | 91.42±5.55 | 93.66±7.30 |
| WHtR | % | 58.88±6.63 | 57.57±6.33 | 60.97±6.44 | 62.23±7.42 |
| BMI | kg/m^2^ | 27.22±4.50 | 26.52±4.45 | 28.39±4.32 | 28.40±4.59 |
| BAI | % | 33.70±4.84 | 32.78±4.57 | 35.20±4.89 | 35.87±5.07 |
| BF | % | 37.11±6.04 | 35.08±5.49 | 40.27±5.23 | 42.96±5.49 |
| FM | kg | 27.66±7.65 | 26.92±7.74 | 29.07±7.38 | 27.84±6.99 |
| FFM | kg | 41.09±4.55 | 41.07±4.61 | 41.33±4.51 | 39.63±3.81 |
| FMI | kg/m^2^ | 10.96±3.01 | 10.51±2.97 | 11.74±2.91 | 11.75±3.08 |
| FFMI | kg/m^2^ | 16.26±1.50 | 16.02±1.49 | 16.67±1.42 | 16.65±1.53 |

Table S3. Pooled Receiver Operating Characteristic (ROC) Analysis of Anthropometric Indices for Prediction of Elevated 10-Year ASCVD Risk (7.5% threshold) in participants Aged 40-70 Years

| Test Result Variable(s) | Area | Std. Error^a^ | P-value^b^ | 95% Confidence Interval |
| --- | --- | --- | --- | --- |
| WHtR | .489 | .028 | .717 | [0.435; 0.543] |
| BF | .556 | .027 | .055 | [0.503; 0.608] |
| WHR | .646 | .026 | .000 | [0.594; 0.698] |
| BMI | .477 | .028 | .430 | [0.422; 0.533] |
| WC | .543 | .028 | .138 | [0.489; 0.598] |
| BAI | .374 | .026 | .000 | [0.324; 0.425] |
| FM | .555 | .028 | .058 | [0.500; 0.610] |
| FMI | .514 | .028 | .625 | [0.460; 0.568] |
| FFM | .526 | .027 | .365 | [0.473; 0.580] |
| FFMI | .387 | .028 | .000 | [0.332; 0.443] |
| LAP | .503 | .029 | .925 | [0.446; 0.560] |
| VAI | .484 | .030 | .587 | [0.426; 0.543] |

WC: waist circumference, WHR: waist-to-hip ratio, WHtR: waist-to-height ratio, HC: hip circumference, BMI: body mass index, BAI: body adiposity index, BF: body fat, FM: fat mass, FFM: fat-free mass, FMI: fat mass index, FFMI: fat-free mass index, LAP: lipid accumulation product, VAI: visceral adiposity index, AUC: Area under the curve.

a. Under the nonparametric assumption

b. Null hypothesis: true area = 0.5

Table S4. Pooled Receiver Operating Characteristic (ROC) Analysis of Anthropometric Indices for Prediction of Elevated 10-Year ASCVD Risk (7.5% threshold) in males Aged 40-70 Years

| Test Result Variable(s) | AUC | Std. Error^a^ | P-value^b^ | 95% Confidence Interval |
| --- | --- | --- | --- | --- |
| WHtR | .566 | .031 | .040 | [0.505; 0.627] |
| BF | .591 | .030 | .005 | [0.532; 0.650] |
| WHR | .606 | .031 | .001 | [0.545; 0.668] |
| BMI | .501 | .032 | .970 | [0.439; 0.546] |
| WC | .518 | .032 | .573 | [0.456; 0.580] |
| BAI | .535 | .031 | .278 | [0.473; 0.596] |
| FM | .508 | .032 | .812 | [0.446; 0.570] |
| FMI | .544 | .031 | .172 | [0.483; 0.605] |
| FFM | .351 | .030 | .000 | [0.291; 0.410] |
| FFMI | .398 | .032 | .002 | [0.336; 0.460] |
| LAP | .493 | .032 | .838 | [0.430; 0.557] |
| VAI | .486 | .033 | .672 | [0.422; 0551] |

WC: waist circumference, WHR: waist-to-hip ratio, WHtR: waist-to-height ratio, HC: hip circumference, BMI: body mass index, BAI: body adiposity index, BF: body fat, FM: fat mass, FFM: fat-free mass, FMI: fat mass index, FFMI: fat-free mass index, LAP: lipid accumulation product, VAI: visceral adiposity index, AUC: Area under the curve.

a. Under the nonparametric assumption

b. Null hypothesis: true area = 0.5

Table S5. Pooled Receiver Operating Characteristic (ROC) Analysis of Anthropometric Indices for Prediction of participants Aged below 40 Years old with high burden cardiovascular risk factors.

| Test Result Variable(s) | AUC | Std. Error^a^ | P-value^b^ | 95% Confidence Interval |
| --- | --- | --- | --- | --- |
| WHR | 0.682 | 0.029 | <0.001 | [0.625; 0.739] |
| BF | 0.728 | 0.027 | <0.001 | [0.674; 0.782] |
| FMI | 0.727 | 0.027 | <0.001 | [0.673; 0.781] |
| WHtR | 0.681 | 0.029 | <0.001 | [0.624; 0.737] |
| WC | 0.747 | 0.026 | <0.001 | [0.695; 0.798] |
| BMI | 0.726 | 0.028 | <0.001 | [0.672; 0.780] |

WC: waist circumference, WHR: waist-to-hip ratio, WHtR: waist-to-height ratio, HC: hip circumference, BMI: body mass index, BAI: body adiposity index, BF: body fat, FM: fat mass, FFM: fat-free mass, FMI: fat mass index, FFMI: fat-free mass index, LAP: lipid accumulation product, VAI: visceral adiposity index, AUC: Area under the curve.

a. Under the nonparametric assumption

b. Null hypothesis: true area = 0.5

Table S6. The frequency results of physical activity levels.

|  | | Frequency | Percent | Valid Percent |
| --- | --- | --- | --- | --- |
| Valid | Low | 215 | 19.4 | 19.4 |
|  | Moderate | 576 | 52.1 | 52.1 |
|  | High | 315 | 28.5 | 28.5 |
|  | Total | 1106 | 100.0 | 100.0 |
